# Repair of bone defect by nano-modified white mineral trioxide aggregates in rabbit: A histopathological study

**DOI:** 10.4317/medoral.20290

**Published:** 2015-06-02

**Authors:** Mohammad-Ali Saghiri, Jafar Orangi, Nader Tanideh, Armen Asatourian, Kamal Janghorban, Franklin Garcia-Godoy, Nader Sheibani

**Affiliations:** 1 Departments of Ophthalmology & Visual Sciences and Biomedical Engineering, University of Wisconsin School of Medicine and Public health, Madison, WI USA; 2 Department of Materials Science and Engineering, School of Engineering, Shiraz University, Shiraz, Iran; 3 Pharmacology Department Shiraz Medical School, Shiraz University of Medical Sciences, Iran; 4 Kamal Asgar research center, Shiraz, Iran; 5 Bioscience Research Center, College of Dentistry, University of Tennessee Health Science Center, Memphis, TN, USA

## Abstract

**Background:**

Many researchers have tried to enhance materials functions in different aspects of science using nano-modification method, and in many cases the results have been encouraging. To evaluate the histopathological responses of the micro-/nano-size cement-type biomaterials derived from calcium silicate-based composition with addition of nano tricalcium aluminate (3CaO.Al2O3) on bone healing response.

**Material and Methods:**

Ninety mature male rabbits were anesthetized and a bone defect was created in the right mandible. The rabbits were divided into three groups, which were in turn subdivided into five subgroups with six animals each based on the defect filled by: white mineral trioxide aggregate (WMTA), Nano-WMTA, WMTA without 3CaO.Al2O3, Nano-WMTA with 2% Nano-3CaO.Al2O3, and empty as control. Twenty, forty and sixty days postoperatively the animals were sacrificed and the right mandibles were removed for histopathological evaluations. Kruskal-Wallis test with post-hoc comparisons based on the LSMeans procedure was used for data analysis.

**Results:**

All the experimental materials provoked a moderate to severe inflammatory reaction, which significantly differed from the control group (*p*< 0.05). Statistical analysis of bone formation and bone regeneration data showed significant differences between groups at 40- and 60- day intervals in all groups. Absence of 3CaO.Al2O3 leads to more inflammation and foreign body reaction than other groups in all time intervals.

**Conclusions:**

Both powder nano-modification and addition of 2% Nano-3CaO.Al2O3 to calcium silicate-based cement enhanced the favorable tissue response and osteogenesis properties of WMTA based materials.

**Key words:**Bone regeneration, cement, endodontics, histopathology, nano-wmta, tricalcium aluminate.

## Introduction

Nano-materials have one or more external dimensions in the size range of 1-100 nm, and are now undergoing rapid development in the field of nanotechnology. Their properties are dependent on their particle size, rendering them superior and indispensable in various fields of research. Nano-sizing effect is involved in various processes and sometimes leads to different behaviors and patterns. It is important to fully grasp biological processes involved at nano levels in order to achieve highest levels of development in the field of nanotechnology (1).

Many types of silicate-based inorganic materials have been reported as osteoconductive materials, such as white mineral trioxide aggregate (WMTA). WMTA is bioactive (2,3), with the capability to form a calcium phosphate layer after immersion in physiological fluids. This bone-like apatite layer can support new tissue formation and its integration into the bone tissue. Thus, WMTA exhibits an essential requirement for an artificial osteoconductive and osteoinductive material. Natural bone surface quite often contains features that resemble nano-material characteristics, which are about 100 nm across. Furthermore, creating nano-sized features on the surface of the hip or knee prosthesis reduces the chance of rejection and stimulate the production of osteoblasts (4). Thus, revealing the impact of micro-/nano-size materials on the living organism function is essential.

The unique physicochemical and biological properties of mineral trioxide aggregate (MTA) might be attributed to its composition and release of Ca2+ and OH ions during hydration (5). The setting mechanism and hydration products of MTA have significant effects on the physiochemical properties of MTA. Studies suggest that a rise in pH induced by MTA, along with the availability of Ca2+ and OH ions, affect mineralization (6). These effects are mainly attributed to the alkaline properties, leading to neutralization of the acidic metabolites of macrophages and osteoclasts. In addition, previous investigations confirmed the desirable effect of alikanie pH on intraosseous responses of MTA implantation (6-8). Apart from these desirable properties, MTA has some disadvantages, including long setting time, weak handling properties and discoloration potential (6,7,9). However, the potential of improving concrete properties by modifying the structure of cement hydrates, addition of nano particles and nano tubes, and controlling the delivery of admixtures have been demonstrated (8,10).

Major ingredients of MTA include tricalcium silicate (3CaO.SiO2), 3CaO.Al2O3, dicalcium silicate (2CaO.SiO2), calcium sulfate dihydrate, and bismuth oxide (Bi2O3) (11). The nano-modified WMTA composition includes (2CaO.SiO2), (3CaO.SiO2), (3CaO.Al2O3), Bi2O3, gypsum, strontium carbonate (SrCO3), zeolite, calcium sulfate (CaSO4), and di-sodium hydrogen phosphate (Na2HPO4) in the range of 40 to 100 nm (8). This nano-modified cement has shown enhanced properties compared other cements (12-16). These additives would affect the chemical reaction of the calcium silicate-based materials with surrounding physiological fluid, as well as the setting behavior of the cement.

Many researchers have tried to overcome the MTA disadvantages by adding some accelerators or additives. While some of these accelerators reduced MTA setting time, they also changed other properties (17,18). However, using one of the WMTA ingredients, such as tricalcium aluminates (3CaO.Al2O3), which has significant role in early stage of WMTA hydration due to its high reactivity with water and production of ettringite (19), might influence physiochemical and biological properties of WMTA, perhaps without changing other properties. In cement paste to form various sulfoaluminate and aluminate phases, 3CaO.Al2O3 may react with gypsum, collectively referred to as ettringite phases (20,21). The ettringite crystals are believed to act as a catalyst for crystal nucleation (20). However, reducing the particle size may influence the properties of materials due to changes in their surface energy and improved the hydration procedure and initial setting time (8). More detail studies regarding kinetic and mechanism of cement hydration could be found elsewhere (21,22).

Many researchers have tried to enhance materials functions in different aspects of science using nano-modification method, and in many cases the results have been encouraging (23,24). Thus, in the present study, we evaluated the histopathological responses of cement-type biomaterials derived from calcium silicate-based composition to describe the micro-/nano-size effects of these materials on bone healing.

## Material and Methods

A total of 90 healthy 6-month-old mature male Dutch rabbits, weighing 2000±200 g, were selected. This study was conducted in accordance with the guidelines and approval of the Animal Ethics Committee of Shiraz University of Medical Sciences #4253. The animal experiments were kept and treated according to recommendations of international standards organization 10993-2, and Helsinki Declaration on animal welfare. The living condition of animal was similar to previous investigations (12), and special care was taken to minimize the injury, pain, and discomfort of the animals at all stages of experiment.

The animals were initially divided into three equal groups and each group was further subdivided into five subgroups with six animals in each subgroup based on the protocol used to fill the defects: A (Nano-WMTA, Patent #US8668770 B2); B (Tooth-colored MTA, Dentsply, Tulsa Dental, Tulsa, OK, USA) considered the standard group; C (Calcium silicate cement similar to WMTA ingredients without 3CaO.Al2O3, Eram New Tech, LLC); and D (Nano-WMTA+ 2% Nano-3CaO.Al2O3, (15-80 nm, Eram New Tech, LLC); the fifth group received no treatment and served as control. The selection of standard and control group was based on ISO 7405.

The animals underwent anesthesia with IM injection of 10% ketamine (Alfasan, Woerden, Netherlands), 44 mg/kg, and 2% xylazine (Bayer, Munich, Germany), 8 mL/kg. Local anesthesia procedure was carried out using an infiltration technique with approximately 0.25 mL of 3% lidocaine. The skin around the ventral aspect of the mandible and neck regions were cleaned of hair and the skin were prepared for an aseptic surgery. An incision measuring 3 cm in length was prepared on the ventral aspect of the mandible and the mandibular symphysis was exposed and accessed. A bone defect was created on the mandible in the diastema space, between the incisor and first molar of each animal with a round carbide bur in a high-speed hand piece under copious irrigation with sterile saline solution. A bone defect was produced on the right mandible, which measured 7×1×1 mm.

Irrigation was carried out with normal saline, bleeding was controlled, the site was dried, and the materials were hand mixed with water (powder/liquid ratio 3:1 by weight) to achieve a puttylike consistency according to manufacturers’ instructions and directly placed in the bony defect. In the control group the bony defects did not receive any materials and were allowed to heal naturally. All the other parameters, including mixing technique, duration of mixing and packing were the same for all the groups. The incisions were sutured with 3-0 silk; flunixin (0.15 mg/Kg), penicillin (22000 IU/Kg) as an analgesic and an anti microbial agent were administered for three days, respectively. The animals received the same diet, in form of soft pellets, and were kept under the same environmental conditions.

The animals were sacrificed at 20-, 40-, and 60-days postoperative interval by being placed in a 70% carbon dioxide chamber for 5-10 minutes. The animal mandibles were then removed and placed in 10% buffered formalin for fixation and then placed in10% formic acid for decalcification. The bony samples were embedded in paraffin blocks. Subsequently, serial sections measuring 6 μm in thickness were cut, and followed by hematoxylin-eosin (H&E) staining. The specimens were evaluated by a blinded pathologist under a light microscope (Zeiss, Goettingen, Germany).

The inflammation was categorized by counting visible inflammatory cells (lymphocytes, plasmacytes, granulocytes and macrophages) under 400X magnification based on studies carried out by Noetzel *et al*. (25), and Panzarini *et al*. (26) as follows:

‒ Grade 0: absence of inflammatory cells

‒ Grade I: Mild inflammation (inflammatory cells < 25)

‒ Grade II: Moderate inflammation (inflammatory cells = 25- 50)

‒ Grade III: Severe inflammation (inflammatory cells = 51- 75)

‒ Grade IV: Abscess formation (inflammatory cells > 75)

Formation of new bone around the implanted materials was evaluated under 100X or 200X magnification as follows (27).

‒ Grade 0: absence of new bone formation

‒ Grade I: slight; presence of bony islets and coverage of less than 25% of the material surface with bone.

‒ Grade II: moderate; coverage of at least 50% of the material surface with bone

‒ Grade III: extensive; complete coverage of the material surface with bone or the formation of an osseous bridge around the material.

Foreign body reaction (aggregation of giant cells around the implanted material) was categorized as follows:

‒ Grade 0: absence of foreign body reaction

‒ Grade I: presence of foreign body reaction

- Statistical analysis

The non parametric nature of data caused Kruskal-Wallis test to be selected for data analysis and post-hoc comparisons were based on the LSMeans procedure in SAS (2002, NC, USA). Level of significance was defined at *p* <0.05.

## Results

**Figure 1:**
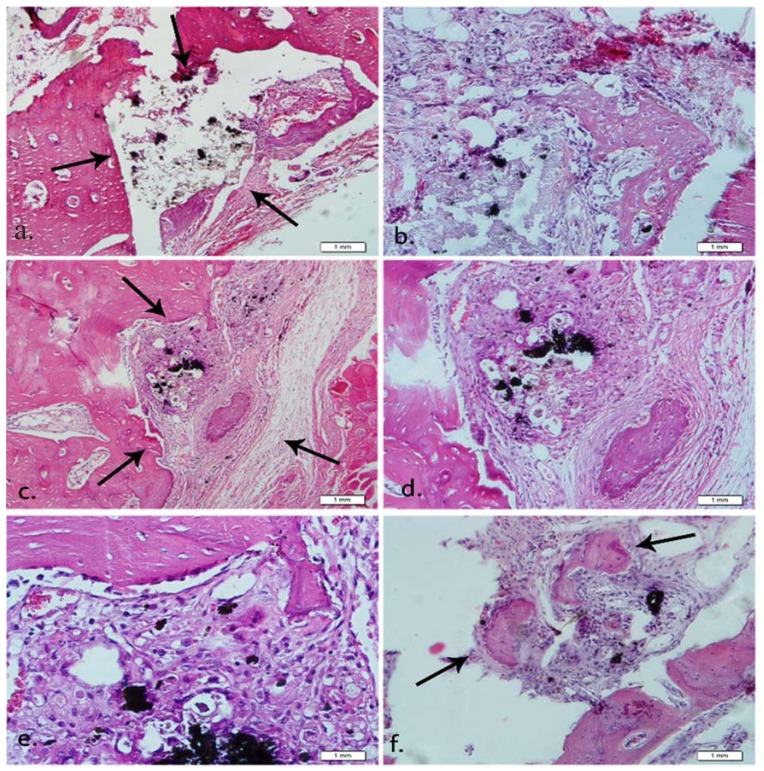
Histopathological images of bone formation, inflammatory cells and foreign body reactions around the experimental materials (hematoxylin and eosin staining). The cement paste is specified with arrows. (a) 40-day group A specimen; grade II in bone formation (original magnification, ×100). (b) 40-day group A specimen; grade II in bone formation (original magnification, ×200). (c) 40-day group C specimen (original magnification, ×100). (d) 40-day group C specimen (original magnification, ×200). (e) 40-day group C specimen; a giant cell can be seen (original magnification, ×400). (f) 40-day group D specimen (original magnification, ×200).

Results of the evaluations including inflammatory response, bone regeneration, and foreign body reaction are summarized in  Table 1. Histopathological images of bone formation are shown in figure 1. The cement paste is specified with arrows.

**Table 1:**
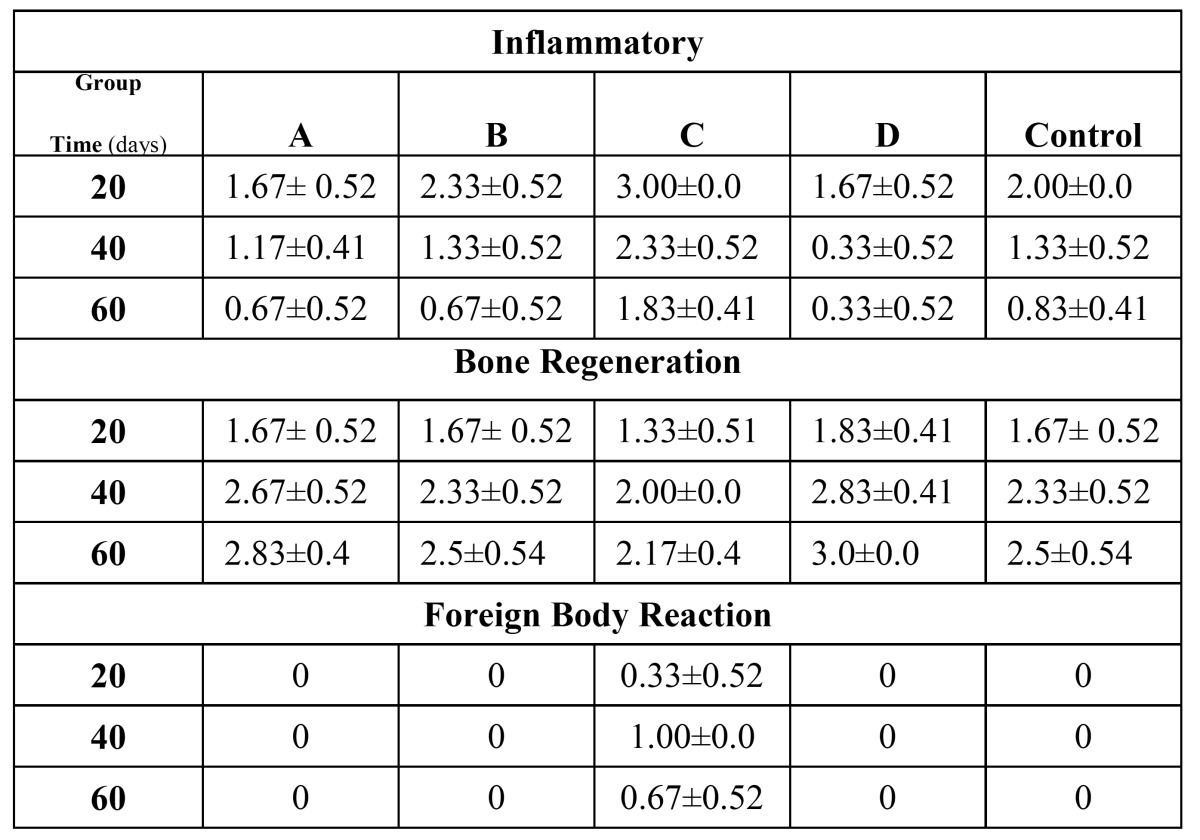
Mean ± SD for inflammatory reaction, bone regeneration and foreign body reaction after the three observation period for different tested materials. Group A, Nano- WMTA; group B, Tooth-colored MTA; group C, Calcium silicate cement; and group D, Nano-WMTA+ 2% Nano-3CaO.Al2O3.

-Inflammatory response

Statistical analysis of inflammatory response using Kruskal-Wallis test showed significant differences between groups at 20-, 40-, and 60-day time intervals (*p*=0.001, *p*=0.001, and *p*=0.004, respectively). Mean ± Standard Deviation (SD) of inflammatory reactions around the experimental materials after 20-, 40-, and 60-days follow up are presented in  Table 1.

-Bone regeneration

Statistical analysis of bone regeneration data using Kruskal-Wallis test showed significant differences between groups at 40-, and 60-day intervals (*p*= 0.041, and *p*= 0.037, respectively). In the 20-day interval there was no significant differences between groups (*p*=0.504). Mean ± SD of bone regeneration around the experimental materials after 20-, 40-, and 60-day follow up is presented in  Table 1.

-Foreign body reaction

Statistical analysis of foreign body reaction using Kruskal-Wallis test showed significant differences between groups at 40-, and 60-days time intervals (*p*<0.001, and *p*= 0.001, respectively). In the 20-day interval there was no significant difference among groups (*p*=0.08). Mean ± SD of foreign body reaction around the experimental materials after 20-, 40-, and 60- day follow up is shown in  Table 1.

## Discussion

This study was an attempt to assess histopathological bone response of WMTA with different particle size, and also with incorporation of nano-particles in a way that it could affect the structure of cement hydrates. In order to improve the biomechanical characteristics of WMTA on surrounding tissue, and at the same time improve its biological and sealing properties, Nano-3CaO.Al2O3 was mixed with the base material. Despite the fact that 3CaO.Al2O3 comprises a small percentage of WMTA chemical composition 3CaO.Al2O3 was selected as a variable due to its undeniable role in WMTA hydration process.

In the present study the inflammatory reaction was decreased at 20-, 40-, and 60-day intervals, which were consistent with the results of previous studies (28,29). The higher inflammation grades in the 20-day samples might be attributed to various factors, including a higher pH value, and release of IL-1 and IL-6 (30).

The 3CaO.Al2O3 is known to reduce the setting time of WMTA since it has the fastest hydration rate among the main components of Portland cement (20). As the material sets, the pH changes and cell injuries subside (31). This could lead to higher inflammatory grades in group C at all-time intervals consistent with the statistical analyses showing significant differences between group C and other groups.

Another variable evaluated in the present study was foreign body reaction, with similar results in all the groups except group C. Foreign body reaction was most evident in group C at 40-day time interval. Longer setting time of group C might be attributed to a shortage of 3CaO.Al2O3, resulting in foreign body reaction.

**Figure 2:**
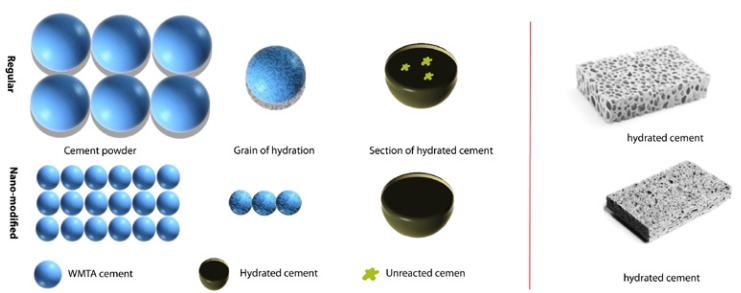
Schematic of particle size effects on hydration reaction. Nano and regular size powder in contact with water, respectively. The bulk of the regular size powder might not react well with water due to low diffusion rate of water molecules through the regular grain size cement.

A method of enhancing the materials property, which is used by many researchers, is to reduce the particle size. Size modification has significant positive effect on materials property (23,24). In cement research the increase in the specific surface area is the main reason for higher reactivity and release of Ca2+ and OH ions. A schematic of the effect of particle size on hydration reaction is presented in figure 2. In the current study, the nano-mixture group has larger surface area than the conventional mixture; therefore, its ability to release Ca2+ and OH ions was increased. These effects might help Nano-WMTA achieve higher pH values in early stages of hydration, resulting in decreased macrophage and osteoclast counts.

Our study suggests that better osteoconductivity can be achieved if synthetic materials resemble bone minerals in composition, size, and morphology as proposed by others (32,33). Hydroxyapatite (HA) crystals in natural bone are nano-size, and may promote the adhesion, proliferation, and synthesis of alkaline phosphates of osteoblasts, leading to a faster repair of hard tissue injuries (34,35). Therefore, nano-modification of WMTA was performed and the results confirmed that Nano-WMTA with 2% Nano-3CaO.Al2O3, and Nano-WMTA had better hard tissue response at all time intervals due to their size, composition, and the ability to release Ca2+ ions, respectively. The results of current study consistent with previous investigation (12), which suggested that bone regeneration was enhanced by reducing the particle size (nano-modified) and tricalcium aluminates mixture. Current study also confirmed that reducing the particle size (nano-modified) and tricalcium aluminates mixture would improve the histopathological reactions in term inflammatory response, bone regeneration, and foreign body reaction.

In the presence of synthetic tissue fluid (STF), MTA precipitated a substance similar to HA. This HA layer is highly biocompatible and has low toxicity. It may also have osteogenic potential because it can release calcium and phosphorus ions, which are involved in bone metabolism (30). In some studies it has been concluded that MTA is catalyzed in the presence of tissue fluids and releases all its cationic content, of which calcium has the highest proportion (36). As a result of nano-modification of WMTA, its specific surface area increased which resulted in composition changes in the vicinity of tissues. The explanation above might be the reason for more bone formation in group D, which resulted in a significant difference from group C in both 40- and 60-day time intervals. The results of tested group materials of current study are consistent with previous studies (12,30,36).

## Conclusion

Within the limitations of the present experiments, it could be concluded that either nano-modified WMTA or addition of 2% Nano-3CaO.Al2O3 induced the most favorable tissue response and osteopromotion properties compared with other tested materials. This was attributed to similarities between bone neutral minerals in composition and size with the tested material, leading to the production of osteoblasts, and its higher pH in early stages of hydration in favor of faster release rate of Ca2+. Although the results are very encouraging, more studies on other properties of these materials are necessary before routine clinical use can be recommended.
